# Score-Level Fusion of 3D Face and 3D Ear for Multimodal Biometric Human Recognition

**DOI:** 10.1155/2022/3019194

**Published:** 2022-04-14

**Authors:** Sumegh Tharewal, Timothy Malche, Pradeep Kumar Tiwari, Mohamed Yaseen Jabarulla, Abeer Ali Alnuaim, Almetwally M. Mostafa, Mohammad Aman Ullah

**Affiliations:** ^1^School of Computer Science, Dr. Vishwanath Karad MIT World Peace University, S. No. 124, Paud Road, Kothrud, Pune 411038, Maharashtra, India; ^2^Manipal University Jaipur, Jaipur, India; ^3^School of Electrical Engineering and Computer Science, Gwangju Institute of Science and Technology, Republic of Korea; ^4^Department of Computer Science and Engineering, College of Applied Studies and Community Services, King Saud University, P.O. BOX 22459, Riyadh 11495, Saudi Arabia; ^5^Department of Information Systems, College of Computer and Information Sciences, King Saud University, P.O. Box 800, Riyadh 11421, Saudi Arabia; ^6^Department of Computer Science and Engineering, International Islamic University Chittagong, Chittagong, Bangladesh

## Abstract

A novel multimodal biometric system is proposed using three-dimensional (3D) face and ear for human recognition. The proposed model overcomes the drawbacks of unimodal biometric systems and solves the 2D biometric problems such as occlusion and illumination. In the proposed model, initially, the principal component analysis (PCA) is utilized for 3D face recognition. Thereafter, the iterative closest point (ICP) is utilized for 3D ear recognition. Finally, the 3D face is fused with a 3D ear using score-level fusion. The simulations are performed on the Face Recognition Grand Challenge database and the University of Notre Dame Collection F database for 3D face and 3D ear datasets, respectively. Experimental results reveal that the proposed model achieves an accuracy of 99.25% using the proposed score-level fusion. Comparative analyses show that the proposed method performs better than other state-of-the-art biometric algorithms in terms of accuracy.

## 1. Introduction

Most biometric systems are unimodal, so they depend on a single modality, i.e., a single source of information is utilized for authentication [1, 2]. In the unimodal biometric framework, there are a few issues such as commotion in sensitive information, entomb class, intraclass varieties, non-all-inclusiveness, and farce assaults. So, we aim to overcome these issues by utilizing multimodal biometrics [3, 4]. The advantage of utilizing multimodal biometrics is that more than one biometric methodology can be fused to provide multiple source data for effective authentication [5, 6].

Three-dimensional biometrics give preferred precision over two-dimensional (2D) biometrics [2, 7]. In addition, 3D biometrics provide more elements and tackle the impediment and brightening issues efficiently. Three-dimensional face gives more highlights than 2D face while handling the issue of impediment and light [8–10]. On the contrary, 3D ear gives more highlights when compared with 2D ear and tackles the issue of impediment [11–13]. The human ear consists of distinct structural features that are fixed with increasing age from 8 to 70 years old with unique highlights. The facial expressions do not affect the ear [14–16].

### 1.1. 3D Face Recognition

Two-dimensional face recognition structures are not capable of solid face affirmation and do not recognize facial images with low light or in poor postures [17, 18]. The research on 3D face recognition techniques is increasing due to the accessibility of advanced 3D imaging devices and their fast computational process. Three-dimensional images of the facial surfaces are acquired for authentication reasons [19–21]. Three-dimensional facial images have a couple of focal points on 2D facial images; it helps to mark straight edges in 3D spacing [22, 23]. The position of 3D facial surfacing relies on the hidden properties of its physical anatomy [24, 25]. The domain of 3D face detection handles the development of techniques for (a) facial identification and (b) verifying people by scanning their 3D facial models [3, 4, 26].

### 1.2. 3D Ear Recognition

Human ears have good characteristics over other biometric modalities: they have a variety of features that are constant between the age group of 8–70 years and ears are not affected by any facial expressions [27–29]. Three-dimensional ear images were proven to be a stable candidate for image recognition as it comes up with three features such as permanence, uniqueness, and universality [5, 6]. However, 3D ear recognition suffers from various problems such as scaling, low illumination, and pose variations.

### 1.3. Challenges and Contributions

Three-dimensional face recognition has many difficulties such as posture variations, facial expressions, aging factors, lighting variations, and image processing methodology. Additionally, due to the large size of images, the computational cost becomes too high than 2D ear recognition models. Also, sometimes these images may contain sparse point clouds which results in low mesh resolution. Thus, to overcome these problems, an efficient fusion-based model is proposed.

The main contributions of this study are as follows:

1. A novel multimodal biometric system is proposed using 3D face and 3D ear for human recognition
2. Principal component analysis (PCA) is utilized for 3D face recognition
3. Independent component analysis (ICA) is utilized for 3D ear recognition
4. Finally, the 3D face is fused with a 3D ear using score-level fusion
5. Extensive experiments are performed by considering benchmark datasets

The remaining paper can be organized as follows.  Section 2 presents the related work.  Section 3 discusses the proposed model.  Section 4 presents the comparative analyses.  Section 5 concludes the study.

## 2. Related Work

We aim to investigate the design of multimodal recognition using ear and facial characteristics. To the best of our knowledge, there have not been several techniques proposed by combining ear and face for biometric recognition. Islam et al. [19] used 3D face and ear features to implement multibiometric human recognition. In this study, feature and score-level fusions were performed by combining the features of 3D face and ear. Iterative closest point (ICP) algorithm was utilized to fuse the scores obtained from fused features. To test the fusion technique, a multimodal dataset was constructed that comprises frontal (FRGC v.2) face and publicly available profile (UND-J) databases. Islam et al. [24] proposed a human recognition system by combining the features of 3D face and ear at the score level. FRGC v.2 and UND-J databases were utilized to evaluate the technique. This technique achieved a 99.68% verification rate and a 98.71% identification rate for fused features.

Nazmeen et al. [20] gave a multibiometric recognition system using the images of the ear and face. The features were extracted using principal component analysis (PCA) (also called Karhunen–Loeve (KL) expansion). The extracted features were then fused at the decision level using the majority vote rule. This technique achieved a 96% recognition rate for fused features. Kyong et al. [25] implemented 3D face recognition using the adaptive rigid multiregion selection (ARMS) technique. It created the fused results by matching the multiple facial regions independently. This technique did not select the landmarks manually; instead, it was fully automatic and achieved 97.5% accuracy. Ajmera et al. [26] proposed improved 3D face recognition using modified SURF descriptors. It achieved recognition rates such as 81.00% and 98.00% on 30° and 15° internal databases, respectively. The recognition rate in the case of EURECOM and CurtinFace databases was achieved as 89.28% and 98.07%, respectively. Hui and Bhanu [27] utilized 3D ear biometrics to implement a human recognition system.

A single reference 3D ear shape model was used for 3D ear detection. A local surface patch (LSP) was used to compute the feature points. For alignment between probe ear and gallery ear, an ICP algorithm was used. Rahman et al. [28] used Krawtchouk moments (KCMs) to implement the face recognition system. Pujitha et al. [29] used Microsoft Kinect to combine the features of face and ear to implement a multimodal biometric system. Contour algorithm and discrete curvelet transform were utilized for ear and face recognitions, respectively. The extracted features were fused at a metric level. Ping and Bowyer [9] used 3D ear shapes to design the biometric recognition system. In this system, segmentation of ear biometrics and matching of 3D ear shape was done. A contour algorithm was used to detect the ear pit. Wu et al. [30] used an edge-based approach and ICP algorithm to implement an ear recognition system. It achieved a 98.8% recognition rate. Algabary et al. [31] implemented ear recognition using stochastic clustering matching (SCM) and ICP. It achieved a 98.25% identification rate. Drira et al. [32] implemented 3D face recognition using a geometric framework. Radial curves were used to represent the facial surfaces. Then, the Riemannian framework was used to analyze these surfaces.

However, the existing techniques suffer from various problems such as overfitting [33–35], generalized model [36, 37], parameters' tuning [38, 39], and poor convergence speed [40, 41]. Therefore, in this study, we have focused on a hybrid model that requires lesser (Table 1) parameters for tuning, no convergence issue, and returns in a generalized model.

## 3. Proposed Model

**Figure 1:**
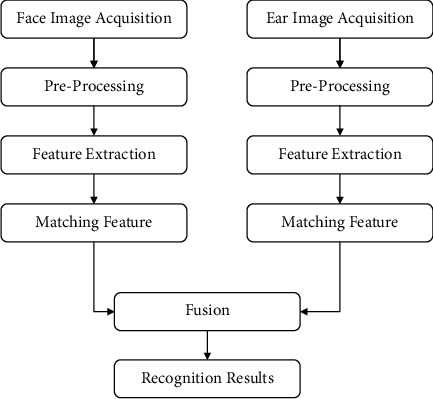
Proposed methodology.

Many researchers convert 3D images into 2D images to perform the 3D face and 3D ear recognition. In the proposed approach, without any conversion, we utilized 3D images for 3D face and 3D ear recognition. In general, researchers considered fewer features for 3D face and 3D ear recognition. However, we have utilized twelve unique features for 3D face and nine unique features for 3D ear recognition. The overall step-by-step flow of the proposed model is shown in Figure 1.

### 3.1. Image Acquisition

We used the Face Recognition Grand Challenge database (FRGC) for 3D face recognition and the University of Notre Dame (UND) Collection F and Collection *G* database for 3D ear recognition. Both the databases were obtained from the University of Notre Dame. They used Minolta vivid 910 scanners to capture the image of 3D face and 3D ear. We used 30 subjects' samples for each 3D face and 3D ear. In the 3D face database, six positions were decided such as anger, disgust, happiness, fear, sadness, and surprise. For the 3D ear database, two ears, i.e., left ear and right ear, with different angles were considered.

### 3.2. Preprocessing

In preprocessing step, first, we read the “.abs” file because we used FRGC 2.0 database for 3D face and Collection F and Collection *G* database for 3D ear. All the images are ASCII text records that have been compressed. The dataset is not unfastened at this time, as the development of these records may necessitate a large amount of plate space. A three-line header appears on each image record and indicates the number of lines and segments. MeshLab tool is also used to open the 3D images. Using this tool, we can directly show and perform the preprocessing operation such as hole filling on 3D images. Nose tip detection is the first step of 3D image preprocessing, and it is detected by using MATLAB software. In the end, the relevant images are cropped. After cropping the desired region, we perform despiking, hole filling, and denoising. For despiking, we use a median filter to remove the spikes and smooth the image.

### 3.3. Feature Extraction

A principal component analysis (PCA) is utilized to extract the features of 3D face. Iterative closest point (ICP) is used to extract the features of 3D ear. Twelve unique features are used for 3D faces such as nose tip, eyes, chin, cheeks, mouth, nostril, nose bridge, eyebrows, four eye corners, two mouth corners, a tip of the chin, and nasal patches. We have used nine unique features for 3D ear recognition such as ear tip, empty center feature, angle feature, point feature, line feature, area feature, curve feature, point cloud, and boundary of points. 3D characteristics are always separated by 3D ear and face datasets. It can be constrained by the distinction between the ﬁrst two eigenvalues in a PCA (concentrated on the key core interests) [16]. The number and regions of the key centers are viewed as various entities for the ear and the face images [17]. At that point, reliably analyzed (on the sliced data centers), a 3D surface of 30 cross segments is used for approximations (using “D'Errico's surface fitting code”). An internal network of 20 × 20 is decomposed to more prominent surface of 400-dimensional vector [19]. Depth map of the nasal region is represented by its point clouds as [22]

(1) $$\begin{array}{c} N=\left[N_{x},N_{y},\;N_{z}\right]. \end{array}$$

The normalized values can be computed as

(2) $$\begin{array}{c} n=\left[n_{x},\;n_{y},n_{z}\right]=\nabla N. \end{array}$$

Here,

(3) $$\begin{array}{c} n_{x}{}^{\circ}n_{x}+n_{y}{}^{\circ}n_{y}+n_{z}{}^{\circ}n_{z}=1. \end{array}$$

Here, *o* and 1 represent the Hadamard product operator and matrix of ones, respectively.

### 3.4. Matching Features

Using the Euclidean distance, features are aligned and matched. It is the most straightforward method of expressing the distance between two places. Euclidean is the length of a segment linking two points in either flat or 3D space, measured by the distance between them. The 3D matching features can be achieved as

(4) $$\begin{array}{c} d\left(p,q\right)\;=\sqrt{{\left(p_{1}-q_{1}\right)}^{2}+{\left(p_{2}-q_{2}\right)}^{2}+{\left(p_{3}-q_{3}\right)}^{2}}. \end{array}$$

Here, *d* is the distance and (*p*, *q*) is the point. Due to the 3D image, the subtraction is done between *p*_1_ and *q*_1_ to *p*_3_ and *q*_3_, respectively.

## 4. Performance Analysis

### 4.1. Datasets

In this study, three different datasets are used. These are discussed in the following sections.

#### 4.1.1. Face Recognition Grand Challenge 2.0 (FRGC)

With 557 subjects, the FRGC dataset is usually regarded as the largest 3D face dataset. The Minolta laser sensor was used to capture the images in the dataset throughout three distinct sessions: Spring 2003, Fall 2003, and Spring 2004. The 3D images were taken under controlled illumination conditions appropriate for the Vivid 900/910 sensor. In FRGC, the 3D images are for both the texture and range channels. Minolta Vivid 900/910 is a structured high sensor that takes a 640 by 480 range sampling and registered colorful images to create the 3D images. The subjects stood or sat around 1.5 meters away from the sensor. For the concern of database standards, Minolta Vivid 910 scanner was used for image acquisition. They captured 466 subjects' data with six different positions such as anger, disgust, happiness, fear, sadness, and surprise. Distance of 1 meter to 1.5 meter was considered with full light illumination having 640 × 480 resolution. The size of the database is 72 GB. [7].

#### 4.1.2. UND Collection F

In this dataset, 942 3D (and corresponding 2D) profile (ear) images from 302 human subjects were captured in 2003 and 2004. Minolta Vivid 910 scanner was utilized for image acquisition, and they captured 466 subjects' data with two different positions. Distance of 1 meter to 1.5 meter was considered with full light illumination having 640 × 480 resolution. The size of the database is 2.5 GB.

#### 4.1.3. UND Collection G

In this dataset, 738 3D (and corresponding 2D) profile (ear) images from 235 human subjects were captured between 2003 and 2005. Minolta Vivid 910 scanner was used for image acquisition, and they captured 466 subjects' data with two different positions. Distance of 1 meter to 1.5 meter is considered with full light illumination having 640 × 480 resolution. The size of the database is 2 GB.

### 4.2. Visual Analyses

**Figure 2:**
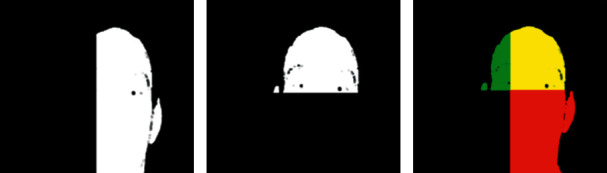
Visual analysis of concatenated coordinates: (a) *X*-coordinate view, (b) *Y*-coordinate view, and (c) concatenation of *X*, *Y*, and Z coordinates.

Figure 2 shows the visual analysis of concatenated coordinates. The *X*-coordinate and *Y*-coordinate views of the image can be seen in Figures 2(a) and 2(b), respectively.  Figure 2(c) shows the concatenation of *X*, *Y*, and *Z* coordinates. It is found that the concatenated 2D view is not showing any kind of details about the 3D face. Thus, it may lead to poor results.

**Figure 3:**
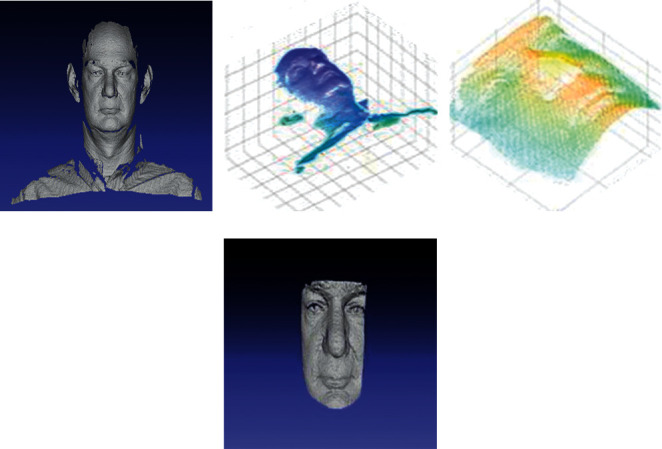
Three-dimensional visualization analyses of 3D face images: (a) 3D face, (b) 3D mesh view, (c) cropped 3D mesh view, and (d) cropped 3D face.

Figure 3 shows the 3D visualization analyses of 3D face images. Figures 3(a) and 3(b) show the 3D face and 3D mesh view, respectively. After applying the various preprocessing operations such as despiking, hole filling, and denoising, a cropped 3D face image is obtained. Figures 3(c) and 3(d) show the cropped 3D mesh view and cropped 3D face image obtained using the preprocessing operations, respectively.

**Figure 4:**
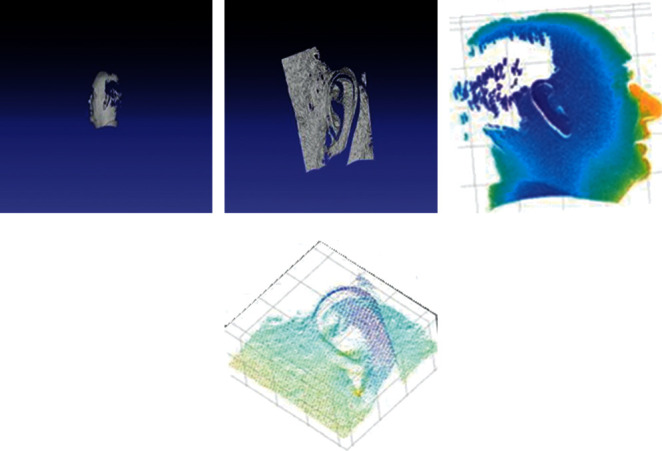
Visual analysis of 3D ear image: (a) 3D ear, (b) 3D mesh view, (c) cropped 3D mesh view, and (d) cropped 3D ear.

Figure 4 shows the 3D visualization analyses of 3D ear images. Figures 4(a) and 4(b) show the 3D ear and 3D mesh view, respectively. After applying the various preprocessing operations such as despiking, hole filling, and denoising, a cropped 3D ear image is obtained. Figures 4(c) and 4(d) represent the cropped 3D mesh view and cropped 3D ear images, respectively.

### 4.3. Quantitative Analyses

**Figure 5:**
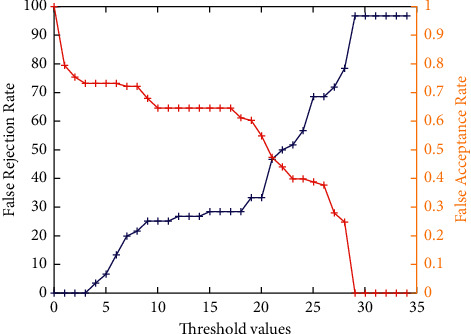
False acceptance rate and false rejection rate analyses of PCA-based 3D face recognition.

**Figure 6:**
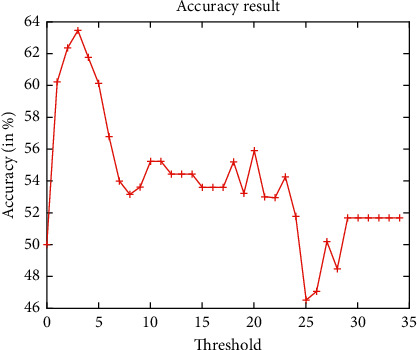
Accuracy analysis of PCA-based 3D face recognition.

Figure 5 shows the false acceptance rate and false rejection rate assessment analyses of the PCA model by considering the obtained cropped PCA-based 3D faces only. It clearly shows that the cropped 3D face images achieve better results. However, for higher threshold values, it achieves poor results. The accuracy analysis of the PCA model by considering the obtained cropped 3D faces only is shown in Figure 6. It is found that, with the increase in threshold values, initially, the performance is increased, but after threshold value 3, it shows a drop in the performance. When a threshold is 25, it is almost 46.24%.

**Figure 7:**
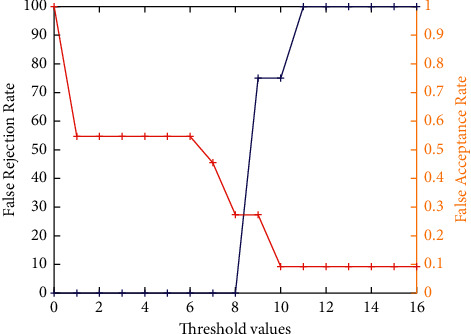
False acceptance rate and false rejection rate analysis of ICP-based 3D ear.

**Figure 8:**
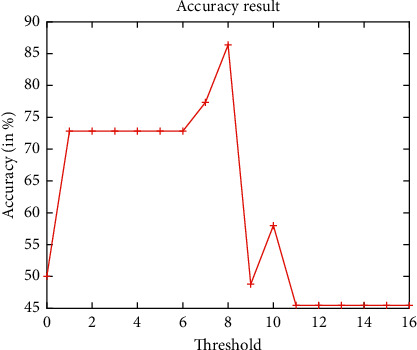
Accuracy analysis of ICP-based 3D ear recognition.

Figure 7 demonstrates the false acceptance rate and false rejection rate assessment analysis of the ICP model by considering the obtained cropped 3D ears only. It clearly shows that the cropped ICP-based 3D ear images achieve better results. However, for higher threshold values, it achieves poor results. The accuracy analysis of the ICP model by considering the obtained cropped 3D ears only is shown in Figure 8. It is found that, with the increase in threshold values, initially, the performance is increased, but after threshold value 8, it shows a decline in the performance.

**Figure 9:**
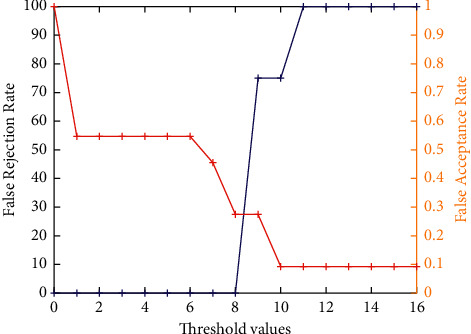
False acceptance rate and false rejection rate analysis of proposed score-level fusion-based 3D ear.

**Figure 10:**
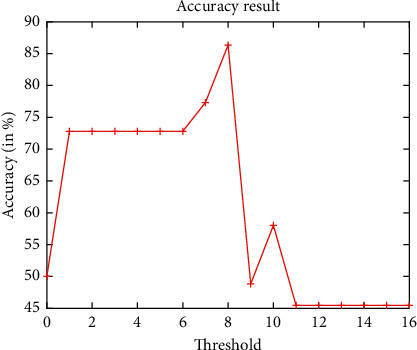
Accuracy analysis of proposed score-level fusion-based 3D ear.

Figure 9 demonstrates the false acceptance rate and false rejection rate assessment analysis of the proposed score-level fusion model by considering the obtained cropped 3D ears only. It clearly shows that the proposed score-level fusion-based 3D ear images achieve better results. It is found that the fusion-based model has achieved remarkable performance than the individual PCA- and ICP-based analysis. The accuracy analysis of the proposed model by considering score-level fusion is shown in Figure 10. It is found that, with the increase in threshold values, initially, the performance is increased, but after threshold value 8, it shows a decline in the performance. Overall, the proposed model has achieved 99.25% accuracy which is significantly better than the competitive models.

Table 2 shows the quantitative analysis of the PCA, ICP, and proposed score-level fusion models. It is found that the PCA-based 3D face recognition model achieves 63.44% accuracy with a threshold of 0.75%. Also, the ICP-based 3D ear recognition model achieves 61.87% accuracy with a threshold of 0.75%. Whereas the proposed model achieves 99.25% accuracy with an equal error rate threshold, i.e., 0.75%.

### 4.4. Comparative Analyses

Table 3 shows the comparative analysis of the proposed model with the state-of-the-art recognition models. In Table 3, dataset size, algorithm, fusion level, and performance of each competitive technique are provided. It can be seen that the proposed score-level fusion model provides high accuracy as compared to other models.

## 5. Conclusion

To overcome occlusion and illumination problems with 2D human recognition, a novel multimodal biometric system was proposed using 3D images. In the proposed model, initially, PCA was utilized for 3D face recognition. Thereafter, ICP was utilized for 3D ear recognition. Finally, the 3D face was fused with a 3D ear using score-level fusion. The simulations were performed on FRGC database for 3D face and UND collection F database for 3D ear. Experimental results revealed that 63.44% accuracy was obtained for a 3D face with a 36.56 error rate threshold. For 3D ear, 86.36% accuracy was obtained with 13.64 error rate threshold. Whereas the proposed score-level fusion model achieved 99.25% accuracy with a 0.75 error rate threshold. Extensive performance analyses revealed that the proposed model achieved an average improvement of 1.2847% over the competitive models.

In the future, deep learning-based 3D face and 3D ear recognition will be designed. We will try to reduce the sensor cost of a 3D scanner by designing an efficient cost-effective 3D scanner. Furthermore, the proposed model will be deployed on lightweight devices such as mobiles and notebooks, for human authentication.

## Figures and Tables

**Table 1:** Comparisons between the state-of-the-art models.

Ref.	Author and year	Biometrics traits	Database	Techniques	Level of fusion	Result (%)
[19]	Islam et al., 2013	3D ear and 3D face	FRGC 2.0Face and UND collection J	ICP	Feature level fusion	96.8
[24]	Islam et al., 2009	3D face and 3D ear	FRGC 2.0Face and UND collection J	ICP	Score level	98.7
[20]	Nazmeen, 2009	Face and ear	Individual	Karhunen–Loeve (KL) expansion, PCA	Decision level	96
[25]	Kyong et al., 2005	2D + 3D face	NA	ARMS	Match score	97.5
[26]	Ajmera, 2014	3D face	EURECOM, CurtinFace, and one internal database	SURF with adaptive histogram equalization	Match score	89.28, 98.07, and 81.00
[27]	Hui and Bhanu, 2007	3D ear	UCR and UND	Local surface patch, global to local registration, and ICP	Rank level	96.77 95.48
[28]	Rahman et al., 2016	Face	FRGC V2.0 and CK-AUC	2D krawtch UK moment(2DKCMs), PCA, LDA, and 2D-PCA	NA	98.70
[29]	Pujitha et al., 2010	Ear and face (2D + 3D)	Captured 2D images using Kinect	Eigenfaces	Feature fusion level	97
[9]	Ping and Bowyer, 2007	3D ear	UND	ICP	Feature level fusion	97.6
[30]	Wu et al., 2012	3D ear	UNDCollection J2	ICP fine alignment	Score level	97.59
[31]	Algabary et al., 2014	3D ear	UND	Iterative closest point and stochastic lustering matching (SCM)	Decision level	98.25
[32]	Drira et al., 2013	3D face	Biosecure residential workshop	ICP	Match score	97.25

**Table 2:** Result analysis of PCA, ICP, and proposed score-level fusion models.

Biometric traits	Techniques	Accuracy	Equal error rate
3D face	PCA	63.44	36.56
3D ear	ICP	86.36	13.64
Fusion	Proposed	99.25	0.75

**Table 3:** Comparative analysis with other approaches.

Model	Data types and size of databases	Algorithm	Fusion level	Performance (%)
Proposed	3D images of 30 persons	PCA and ICP	Score	99.25
Nazmeen et al. [20]	420 images of 30 persons	KL and PCA	Decision	96
Xu and mu [6]	190 images of 38 persons	KCCA	Feature	98.7
Xu et al. [4]	2D images of 79 persons	KFDA	Feature	96.8
Wu et al. [30]	3D images of 415 persons	ICP	Score	97.59
Chang et al. [3]	197 2D images	PCA	Data	90.9
Islam et al. [24]	3D images of 326 persons	L3DFs and ICP	Score	98.1

## Data Availability

The data used to support the findings of this study are available from the corresponding author upon request.
